# Supplementary material for: T Cells Expressing a TCR-Like Antibody Selected Against the Heteroclitic Variant of a Shared MAGE-A Epitope Do Not Recognise the Cognate Epitope
Source: Cancers (Basel). 2020 May 16;12(5):1255. doi: 10.3390/cancers12051255 (PMC7281252; doi:10.3390/cancers12051255)
Supplement: Supplementary file 1 [file cancers-12-01255-s001.pdf]

cancers

Supplementary Materials

T Cells Expressing a TCR-Like Antibody Selected Against the Heteroclitic Variant of a Shared MAGE-A Epitope Do Not Recognise the Cognate Epitope

Mesha Saeed, Erik Schooten, Mandy van Brakel, David K. Cole, Timo L. M. ten Hagen* and Reno Debets

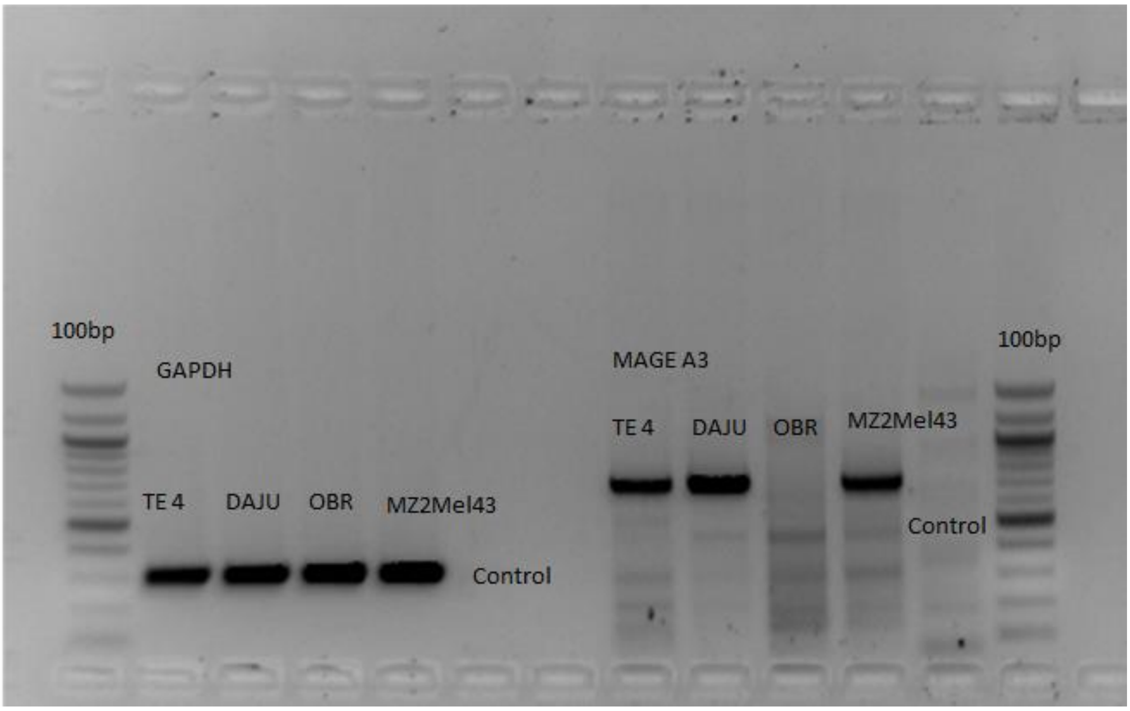

Figure S1. Complete PCR Gel showing the expression of MAGE A3 on tumor cell lines. GAPDH taken along as a control.

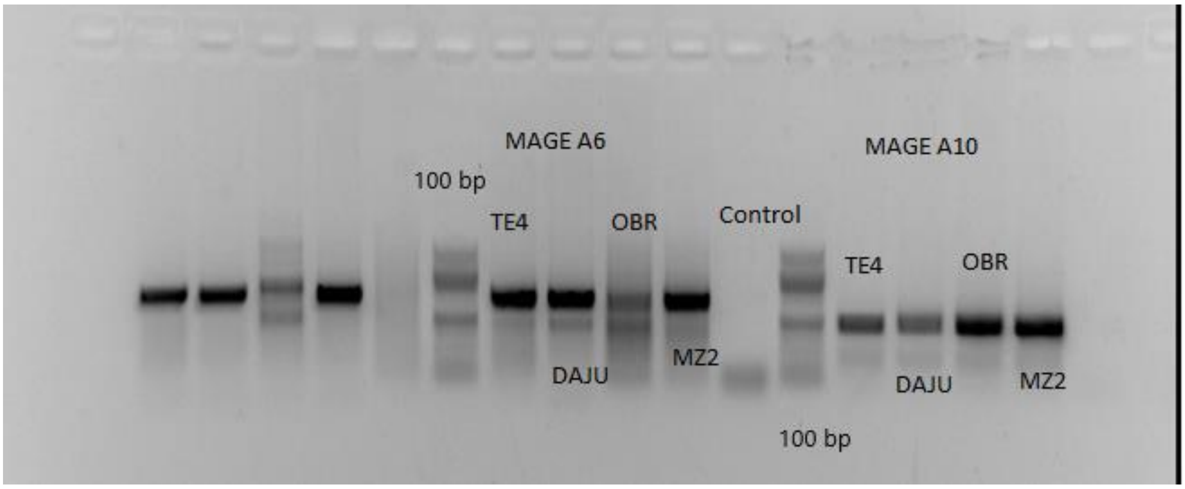

Figure S2. Complete PCR Gel showing the expression of MAGE A6 and MAGE A10 on tumor cell lines.

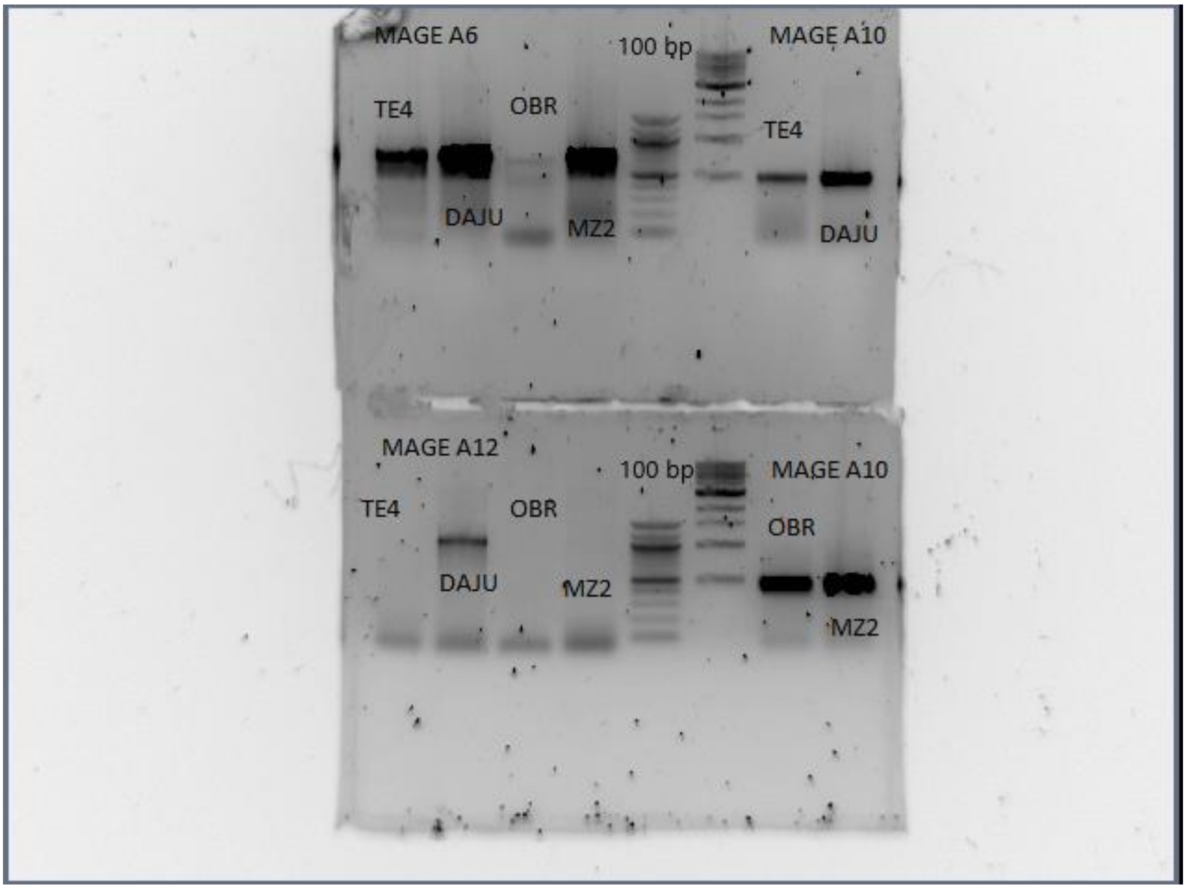

Figure S3. Complete PCR Gel showing the expression of MAGE A6, MAGE A10 and MAGE A12 on tumor cell lines.

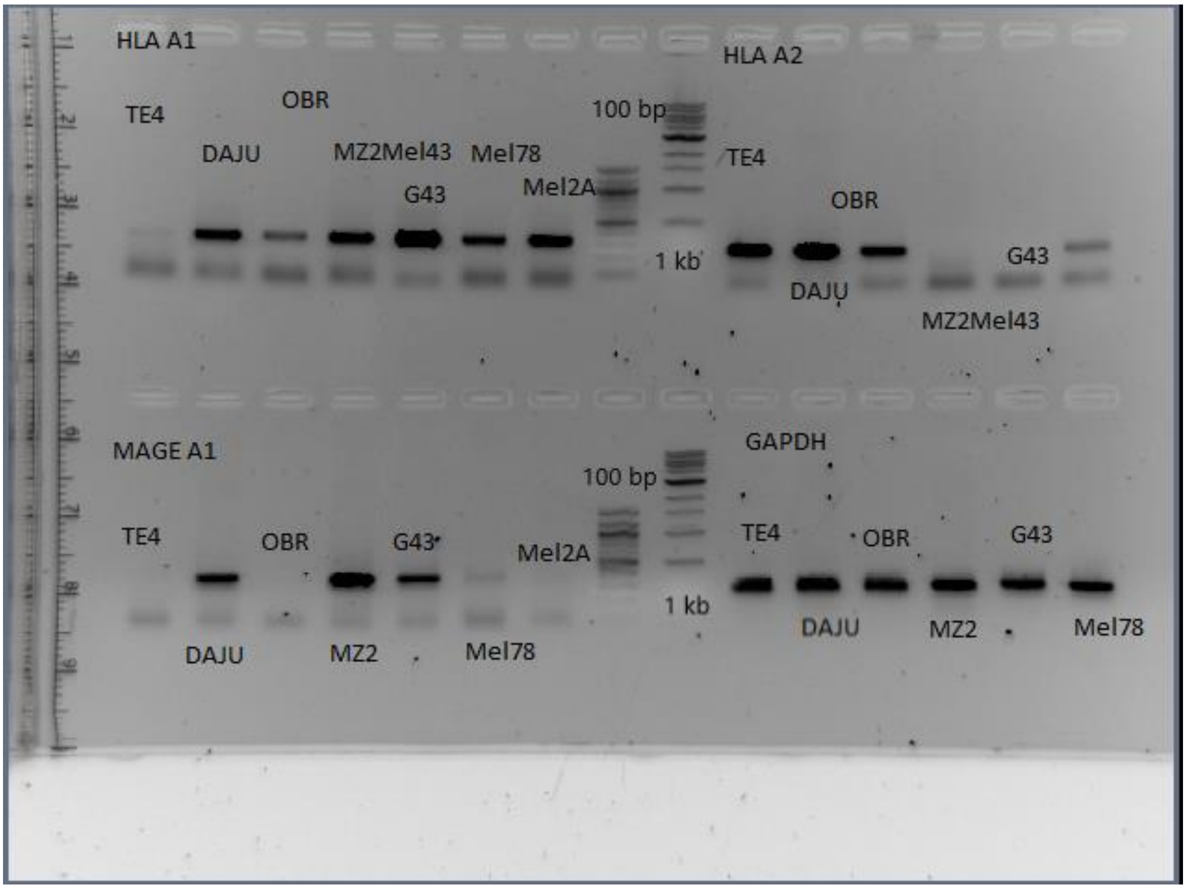

Figure S4. Complete PCR Gel showing the expression of MAGE A1, HLA A1, HLA A2 and GAPDH on tumor cell lines.

Table S1. Sizes and compositions of the phage-Fab libraries.

Antibody source[a]	Library	Insert (%)[b]	Size (cfu)[c]
VH or VL libraries			
PBL	VH	60	$5.6x10^8$
	Vκ	95	$2.0x10^8$
	Vλ	90	$2.9x10^8$
Spleen	VH	99	$1.2x10^7$
	Vκ	100	$4.0x10^7$
	Vλ	90	$5.2x10^7$
Fab libraries			
PBL	VH^{PBL} VL^{PBL}	80	$1.4x10^9$
Spleen	VH^{Spleen} VL^{Spleen}	75	$4.2x10^9$
combi Fab libraries			
PBL/ Spleen	VH^{PBL} VL^{Spleen}	78	$2.3x10^9$
Spleen/ PBL	VH^{Spleen} VL^{PBL}	95	$6.5x10^8$
Total size[d]		82	$8.6x10^9$

[a]See the MM section for a description of the different (combinatorial) libraries. [b]Percentages of inserts determined by PCR using a minimum of 20 clones per library. [c]Sizes of libraries given in clone forming units (cfu) and not corrected for clones without a Fab insert. [d]Sum of cfu's of both Fab libraries and combinatorial libraries.

Table S2. Validation of phage-Fab library following selections with a set of control proteins.

Antigen	Selection round	Binders (ELISA)	Unique clones (restriction patterns)
NEF[a]	2	71/94	nt
LMP1[b]	3	72/94	6/72
PSMA[c] (EKM)	3	39/93	6/39

[a] NEF_{56-205} (CAA13474.1): negative regulatory factor; nt is not tested. [b] $LMP1_{374-386}$ (QAR15098.1): latent membrane protein 1. [c] PMSA $(EKM)_{307-750}$ (AAA60209.1): prostate specific membrane antigen.

Table S3. Sequences of control proteins used for validation of phage-Fab library.

**NEF_{56-205}**<br>(NCBI Genbank acc. number: CAA13474.1)	AWLEAQEEEGDVGFPVRPQVPLRPMTYKGAFDLGFFLKEK<br>GGLDGLVYSKKRQEILDLWVYHTQGFFPDWQCYTPGPGVR<br>YPLTFGWCYKLVPLDPTEVEEANKGENNSLLHPICQHGMDD<br>EDGEVLVWRFDSSLARRHVARELHPEFYK
**$LMP1_{374-386}$**<br>(NCBI Genbank acc. number: QAR15098.1)	DDPHGPVQLSYYD
**$PSMA_{307-750}$**<br>(NCBI Genbank acc. number: AAA60209.1)	EKMGGSAPPDSSWRGSLKVPYNVGPGFTGNFSTQKVKMHI<br>HSTNEVTRIYNVIGTLRGAVEPDRYVILGGHRDSWVFGGIDP<br>QSGAAVVHEIVRSFGTLKKEGWRPRRTILFASWDAEEFGLLG<br>STEWAEENSRLLQERGVAYINADSSIEGNYTLRVDCTPLMYSL<br>VHNLTKELKSPDEGFEGKSLYESWTKKSPSPEFSGMPRISKLGS<br>GNDFEVFFQRLGIASGRARYTKNWETNKFSGYPLYHSVYETYE<br>LVEKFYDPMFKYHLTVAQVRGGMVFELANSIVLPFDCRDYAV<br>VLRKYADKIYSISMKHPQEMKTYSVSFDSLFSAVKNFTEIASKF<br>SERLQDFDKSNPIVLRMMNDQLMFLERAFIDPLGLPDRPFYR<br>HVIYAPSSHNKYAGESFPGIYDALFDIESKVDPSKAWGEVKRQ<br>IYVAAFTVQAAAETLSEVA

NEF: negative regulatory factor; LMP1: latent membrane protein 1; PMSA (EKM): prostate specific membrane antigen.
